# Guidelines for fracture risk assessment and management of osteoporosis in postmenopausal women and men above the age of 50 in Qatar

**DOI:** 10.1007/s11657-024-01389-0

**Published:** 2024-05-02

**Authors:** Fiaz Alam, Omar Alsaed, Nabeel Abdulla, Ibrahim Abdulmomen, Abdo Lutf, Samar Al Emadi

**Affiliations:** Rheumatology Section, Department of Medicine, Hamad Medical Corporation, Doha, Qatar

**Keywords:** Osteoporosis, Fragility fractures, Guidelines, Fracture risk assessment tool, FRAX

## Abstract

**Summary:**

We present comprehensive guidelines for osteoporosis management in Qatar. Formulated by the Qatar Osteoporosis Association, the guidelines recommend the age-dependent Qatar fracture risk assessment tool for screening, emphasizing risk-based treatment strategies and discouraging routine dual-energy X-ray scans. They offer a vital resource for physicians managing osteoporosis and fragility fractures nationwide.

**Purpose:**

Osteoporosis and related fragility fractures are a growing public health issue with an impact on individuals and the healthcare system. We aimed to present guidelines providing unified guidance to all healthcare professionals in Qatar regarding the management of osteoporosis.

**Methods:**

The Qatar Osteoporosis Association formulated guidelines for the diagnosis and management of osteoporosis in postmenopausal women and men above the age of 50. A panel of six local rheumatologists who are experts in the field of osteoporosis met together and conducted an extensive review of published articles and local and international guidelines to formulate guidance for the screening and management of postmenopausal women and men older than 50 years in Qatar.

**Results:**

The guidelines emphasize the use of the age-dependent hybrid model of the Qatar fracture risk assessment tool for screening osteoporosis and risk categorization. The guidelines include screening, risk stratification, investigations, treatment, and monitoring of patients with osteoporosis. The use of a dual-energy X-ray absorptiometry scan without any risk factors is discouraged. Treatment options are recommended based on risk stratification.

**Conclusion:**

Guidance is provided to all physicians across the country who are involved in the care of patients with osteoporosis and fragility fractures.

## Introduction

Osteoporosis is a systemic skeletal disease characterized by low bone mass and microarchitectural deterioration that makes the bone more fragile and prone to fracture [1]. Osteoporosis is a major public health issue with significant implications for both individuals and countries as a whole. Healthcare costs associated with the management of osteoporosis and fragility fractures are substantial, increasing the burden on healthcare systems.

Here, we present guidelines developed by the rheumatology department under the umbrella of Qatar Rheumatology Association of the Hamad Medical Corporation. We believe they are essential tools in healthcare to standardize and improve the quality of patient care. The primary goal was to improve the overall efficacy of the diagnosis and management of patients with or at risk of osteoporosis. These guidelines are focused on treatment of patients at high risk for osteoporotic fracture rather than prevention of osteoporosis. This implies a focus on enhancing the accuracy and effectiveness of healthcare interventions related to osteoporosis.

## Methods

The Qatar guidelines for fracture risk assessment and management of osteoporosis were adapted from international and regional guidelines [2–6]. A group of six local rheumatologists met on the 13th of August 2020 to develop guidance for formulating guidelines for osteoporosis management in Qatar. This kick-off meeting has been followed by several meetings by the local team for building up Qatar fracture risk assessment tool (FRAX) and reviewing and adapting the international and regional guidelines to fit both the governmental and private health systems in Qatar. The rationale for osteoporosis diagnosis has changed dramatically over the last 20 years. Mostly, the initiation of osteoporosis treatment was based on bone mineral density (T-score less than − 2.5), which is, according to the World Health Organization, definition of osteoporosis. Now, most, if not all, international guidelines are in favor of initiating treatment based on the individual’s 10-year probability of fracture risk, rather than using the T-score solely. To cope with the shift in diagnosis, formulating Qatar guidelines went into two stages. The first step was to build up the Qatar FRAX® model, which was based on local epidemiological data on osteoporotic hip fractures [7]. Qatar FRAX went live in October 2021, and now it is accessible (https://www.sheffield.ac.uk/FRAX/tool.aspx?country=85, https://fraxplus.org/)) for all professionals involved in osteoporosis management [8]. The second step was extensive reviewing and adapting the international and regional guidelines to come up with a local guideline that meets the local needs and to make the best use of available resources for the governmental and local healthcare institutes.

### Fracture risk assessment

#### Identifying clinical risk factors associated with fragility fracture

A comprehensive history review and physical examination are essential to identifying clinical risk factors associated with fragility fractures.

1. Bone mineral density-independent- and FRAX accommodated clinical risk factors

(a) Height and weight (body mass index, BMI)

(b) A history of a fragility fracture (excluding hands, feet, skull, and face)

(c) A parental history of hip fractures before the age of 80 years [9]

(d) Active smoking

(e) Systemic glucocorticoids of daily use for more than 3 months

(f) Alcohol intake of 3 units or more per day. The risk increases proportionally with alcohol dosage

(g) Rheumatoid arthritis. Rheumatoid arthritis increases fracture risk independently of bone mineral density (BMD) and the use of glucocorticoids

2. BMD-independent and non-accommodated FRAX clinical risk factors

(a) Recurrent falls and factors that increase the risk of falls, such as vision impairment, neuromuscular disorders (stroke, peripheral neuropathy, Parkinson’s disease), dementia, orthostatic hypotension, and poor home accessibility.

(b) Recency, site, number of previous fragility fractures, and patient’s age at the time of the first fracture.

- The fracture risk after the first fragility fracture is the highest in the first 2 years.
- In the absence of other risk factors, a primary fracture at a younger age (e.g., in their 50s) is associated with a higher risk of a secondary fracture than is a primary fracture at an advanced age (e.g., in their 80s or 90s). This is because the risk of death is competing with the 10-year probability of having a secondary fracture.
- Among major osteoporotic fractures (MOFs), developing secondary fractures is the most common in post-spine fractures and the lowest in post-distal forearm fractures.

(c) Chronic use of a medium or high glucocorticoid dose (> prednisolone 7.5 mg daily or equivalent) for more than 3 months.

(d) Diabetes mellitus type II. The latest information suggests that diabetes may also exert BMD-independent effects on fracture risk.

FRAX adjustment tools for some of these non-accommodated FRAX factors will be elaborated on under Qatar FRAX headlines.

3. BMD-dependent or partially dependent clinical risk factors

(a) Secondary causes: type I (insulin dependent) diabetes, osteogenesis imperfecta in adults, untreated long-standing hyperthyroidism, hypogonadism, or premature menopause (< 45 years), chronic malnutrition or malabsorption, and chronic liver disease. These conditions are part of the FRAX. Their impact on FRAX will be negligible when BMD values are added to FRAX.

(b) Other medical conditions that are not part of the FRAX. Celiac disease, cystic fibrosis, sickle cell disease, rheumatological diseases other than rheumatoid arthritis, IBD (inflammatory bowel disease), advanced obstructive lung diseases, renal disease, drug history: androgen deprivation agents, estrogen deprivation agents (aromatase inhibitors), proton pump inhibitors, selective serotonin reuptake inhibitors (SSRIs), thiazolidinediones, anti-parkinsonian, and anticonvulsants. The mechanism by which some of these factors contribute to predisposing fractures are not well established. Whether it is through impacting BMD, bone architecture, or increasing the risk of falls is still unknown. Table 1 summarizes clinical risk factors associated with fragility fractures.

**Table 1:** Risk factors associated with fragility fractures

BMD-independent risk factors		BMD-dependent or partially dependent
Accommodated for in FRAX	Not accommodated for in FRAX^c^	
Advancing in age Prior fragility fracture Parental hip fracture Height and Weight (body mass index, BMI) Current smoking Rheumatoid arthritis Glucocorticoids 2.5–7.5 mg Alcohol intake of 3 units/day	Characters of prior MOF -Fractures < 2 years -Number of fractures -Site of the fracture -Individual’s age at the time of fracture Glucocorticoids > 7.5 mg/day Recurrent falls Diabetes mellitus type II	Endocrine causes -Diabetes mellitus type I -Untreated long-standing hyperthyroidism -Hypogonadism -Premature menopause (<45 years) -Osteogenesis imperfecta Malabsorption conditions (celiac’s disease, cystic fibrosis, IBD, bariatric surgical procedures) Chronic liver disease Chronic kidney disease Commonly used drugs^a^ -Estrogen deprivation products; aromatase inhibitor (Letrozole, Anastrozole, Exemestane). -Androgen deprivation drugs -Others^b^: proton pump inhibitors, anticonvulsants, antipsychotic, antiparkinsonian disease

*FRAX* fracture risk assessment tool, *BMD* bone mineral density, *MOF* major osteoporotic fracture

^a^We mentioned here the list of medications that are available in Qatar; however, there are many other drugs that could have an impact on fracture risk but are currently not available in Qatar

^b^The impact of some of these drugs on BMD is not well established

^c^FRAX adjustments for these factors are shown under the Qatar FRXA headline

#### Qatar FRAX

Country-specific FRAX calculator for Qatar is now available online since 2021.

Age-specific intervention thresholds for the 10-year risk of MOF and hip were derived in Qatar FRAX modal based upon a woman with prior fracture, BMI of 30 kg/m^2^, and no other clinical risk factors for fracture. Age-dependent hybrid interventional threshold modal of FRAX is recommended to use in which the interventional threshold is set at a level equivalent to the risk of fracture for a woman of the same age who has previously experienced a fracture, till the age of 70 and a fixed interventional threshold is set after the age of 70 [8]. It should be used for all Qatari individuals (citizens) aged 40 and above. For expatriates who are residing in Qatar, it is recommended to use the one that belongs to their original country. If it is not available, then it is recommended to use the closest geographically available national FRAX calculator.

After identifying all clinical risk factors associated with an increased risk of developing fragility fractures, the next step is to feed these variables into the FRAX tool. It is well known that FRAX has many limitations [10]. To overcome these limitations, it is advisable to use FRAXplus whenever accessible (https://fraxplus.org/) or make the following adjustments for the current FRAX.

### FRAX adjustment for glucocorticoid dose

For patients who need to take medium or high doses of glucocorticoid for more than 3 months (prednisolone dose > 7.5 mg daily or equivalent), MOF probability should be increased by 15% and hip fracture probability by 20% (this means multiplying the MOF probability by 1.15 and hip fracture by 1.2) [11]. Appropriate glucocorticoid replacement in individuals with adrenal insufficiency has not been demonstrated to increase fracture risk. In such patients, the use of glucocorticoid should not be included in FRAX calculations.

### FRAX adjustment for recurrent falls

A report from the International Society for Clinical Densitometry (ISCD)/International Osteoporosis Foundation (IOF) Task Force recommended that FRAX probability may be modified to account for a history of prior falls (two or more falls in a year), with the output inflated by 30% (multiplied by 1.3) [12]. Falls adjustment is available via FRAXplus as well [13].

### FRAX adjustment for recent fracture

Developing a secondary fracture is the highest in the first 2 years. For example, a prior clinical vertebral fracture within the past 2 years in a woman aged 70 years is associated with a 1.52-fold higher major fracture probability than is a prior fragility fracture of uncertain recency in a woman of the same age [14, 15].

### FRAX adjustment when there is a discordance between BMDs of lumbar spine and hip

For every rounded 1-unit T-score difference between BMDs of the lumbar spine and hip, there will be an increase/decrease in the MOF probability by 10% [16]. For instance, if the T-score for the lumbar spine is − 4.2 and that of the hip is − 2.2, then the risk of MOF will be increased by 20%, which in turn means multiplying MOF probability by 1.2.

### FRAX adjustment for trabecular bone score

It is recommended to adjust FRAX probability with trabecular bone score (TBS) whenever it is available for patients with BMIs (body mass index) ranging from 15 to 35 kg/m^2^ [17]. FRAX-adjusted TBS is a computerized Web-based adjustment, and it is available for all professionals managing osteoporosis. It has been evident that TBS has the capability of predicting fragility fractures independently of the BMD. Furthermore, TBS can estimate the impact of some medical conditions (diabetes mellitus, hyperparathyroidism, HIV, oncological conditions) [18–25] and drugs (glucocorticoids, aromatase inhibitors) [26–30] on predisposing fragility fractures. Dual-energy X-ray absorptiometry (DXA), FRAX and TBS complement each other to have an optimal fracture risk assessment.

### FRAX adjustment in patients with diabetes mellitus type II

Four proposed methods to improve the performance of FRAX for type II diabetes mellitus:

- Including the rheumatoid arthritis input to FRAX
- Making a TBS adjustment to FRAX
- Reducing the femoral neck T-score input to FRAX by 0.5 SD
- Increasing the age input to FRAX by 10 years [31]

### FRAX adjustment for sex hormone deprivation products users

In women with breast cancer treated with aromatase inhibitors or men with prostate cancer receiving androgen deprivation therapy, FRAX should be adjusted by selecting rheumatoid arthritis as an equivalent risk [32].

#### Bone mineral density measurement

Below are BMD measurement recommendations for all professionals involved in osteoporosis management:

-Areal bone mineral density (aBMD), measured by DXA scan, is the gold standard tool for quantitative bone assessment.

-BMD scan request is recommended for individuals who crossed the lower assessment threshold of FRAX either for MOF or hip fractures, and for monitoring purposes for individuals who will be commencing osteoporosis medications. Routine BMD scanning for individuals who do not have any risk factors or are not based on the FRAX assessment is not recommended.

-The US Third National Health and Nutrition Examination Survey (NHANES III) of white young (ages 20–40) female reference curve is recommended for calculating the T-score for lumbar spine, neck of femur, hip, and forearm in both female and male patients.

-BMD scanning, reading, and interpreting DXA reports should be performed by trained technologists and physicians according to the ISCD positions. Vertebral fracture assessment is advisable for all individuals who undergo routine DXA scan.

#### Osteoporosis screening and case identification

Currently, there is no approved tool in Qatar for population-based screening of osteoporosis. We recommend stratifying individuals according to their verified risk factors for fragility fractures. Primary health care physicians should use, at a minimum, FRAX tool in all individuals aged ≥ 40 years to identify patients with a high risk profile for fractures and refer them to a specialized osteoporosis clinic for management and follow-up. For individuals who have a low risk profile, it is recommended to re-FRAX them whenever there are changes in their clinical profile. DXA scan should be restricted to individuals whose FRAX cross the lower assessment threshold either for MOF or hip fractures. Clinical risk factors which are not accommodated in the FRAX tool should also trigger clinicians to perform an evaluation of an individual’s bone health profile, including DXA. For instance, patients with chronic inflammatory conditions, hemoglobinopathies, lymphoproliferative disease, potential organ transplants or post-transplant recipients, frequent fallers, kyphotic patients, patients receiving medications known to affect bone health or having any other conditions known to have a negative impact on bone quantity or quality. Initiating treatment in this special population will be left to the clinician’s judgment. A routine DXA scan based solely on individual age is not recommended.

## Recommendations

### Screening

*Q1. How can I screen my patients for the risk of having osteoporotic fracture?*

The FRAX is recommended as a screening tool for assessing osteoporotic fracture risk [4, 6, 33–38]. All post-menopausal women and men older than 50 years should be screened.

FRAX is widely used for the estimation of an individual’s 10-year probability of having a MOF or a hip fracture. FRAX takes into account multiple independent risk factors for osteoporotic fracture, and it provides an estimate of absolute fracture risk even in the absence of BMD measurements. The effectiveness of FRAX was evaluated in a randomized control trial, demonstrating that screening with the FRAX tool decreased the incidence of hip fracture in older women [39]. The age-dependent hybrid FRAX model should be used for all Qatari citizens. For expatriates who are residing in Qatar, it is recommended to use the one that belongs to their original country. If it is not available, then it is recommended to use the closest geographically available FRAX calculator.

A previous fragility fracture is indicative of increased bone fragility and an elevated risk of future fractures.

Women who have already experienced a fragility fracture are considered to be at an increased risk, and most guidelines recommend treatment without the need for a BMD test.

Accordingly, the intervention threshold for treatment is developed at the age-specific fracture probability equivalent to women with a prior fragility fracture. This approach is validated and used in many countries around the world. The use of age-specific thresholds in FRAX will guide clinicians in deciding when to request BMD testing and how to categorize patients into low, high, or very high risk for fracture in order to choose the best treatment plan for each individual patient. Following are the different thresholds that will guide clinicians in the assessment of osteoporosis (Table 2).

- The intervention threshold (IT) is the probability of osteoporotic fracture for a woman with a BMI of 30 kg/m^2^ and a previous fracture and no other clinical risk factors without adding any BMD value.
- The lower assessment threshold (LAT) is the probability of osteoporotic fracture for a woman with a BMI of 30 kg/m^2^ and no clinical risk factors without a BMD value.
- The upper assessment threshold (UAT) is set at 1.2 times the intervention threshold (20% increased risk) without adding BMD value.

**Table 2:** Age-dependent LAT, IT, and UAT values

Age	LAT		Intervention threshold		UAT	
	MOF	Hip fracture	MOF	Hip fracture	MOF	Hip fracture
40	1.63	0.05	3.62	0.26	4.34	0.31
41	1.67	0.05	3.7	0.27	4.44	0.32
42	1.7	0.06	3.74	0.29	4.49	0.35
43	1.73	0.06	3.81	0.31	4.57	0.37
44	1.75	0.07	3.84	0.32	4.61	0.38
45	1.81	0.07	3.94	0.34	4.73	0.41
46	1.84	0.08	4.0	0.36	4.80	0.43
47	1.87	0.09	4.05	0.38	4.86	0.46
48	1.89	0.09	4.08	0.4	4.90	0.48
49	1.9	0.1	4.1	0.42	4.92	0.50
50	1.9	0.11	4.09	0.45	4.91	0.54
51	1.9	0.12	4.07	0.48	4.88	0.58
52	1.89	0.14	4.06	0.52	4.87	0.62
53	1.91	0.15	4.08	0.56	4.90	0.67
54	1.93	0.17	4.12	0.61	4.94	0.73
55	1.97	0.2	4.2	0.67	5.04	0.80
56	2.03	0.22	4.31	0.73	5.17	0.88
57	2.11	0.25	4.47	0.8	5.36	0.96
58	2.21	0.29	4.66	0.89	5.59	1.07
59	2.33	0.33	4.89	0.98	5.87	1.18
60	2.47	0.37	5.16	1.08	6.19	1.30
61	2.62	0.42	5.44	1.19	6.53	1.43
62	2.8	0.48	5.77	1.3	6.92	1.56
63	3.0	0.54	6.12	1.43	7.34	1.72
64	3.21	0.61	6.5	1.57	7.80	1.88
65	3.43	0.69	6.89	1.72	8.27	2.06
66	3.67	0.78	7.3	1.89	8.76	2.27
67	3.92	0.89	7.73	2.06	9.28	2.47
68	4.19	1.0	8.17	2.26	9.80	2.71
69	4.46	1.13	8.62	2.47	10.34	2.96
70-90	4.75	1.27	9.06	2.69	10.87	3.23

*IT* intervention threshold, *LAT* lower assessment threshold, *UAT* upper assessment threshold, *MOF* major osteoporotic fracture

It is pertinent to note that a hybrid model is used where an age-dependent threshold is set until the age of 70 and a fixed threshold is used for patients older than 70 years.

**Fig. 1:**
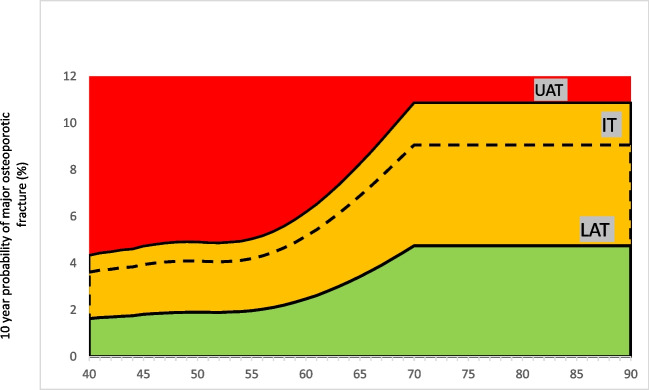
Age-dependent 10-year probability of major osteoporotic fracture (MOF), with the cutoffs for LAT, UAT, and IT. IT, intervention threshold; LAT, lower assessment threshold; UAT, upper assessment threshold

**Fig. 2:**
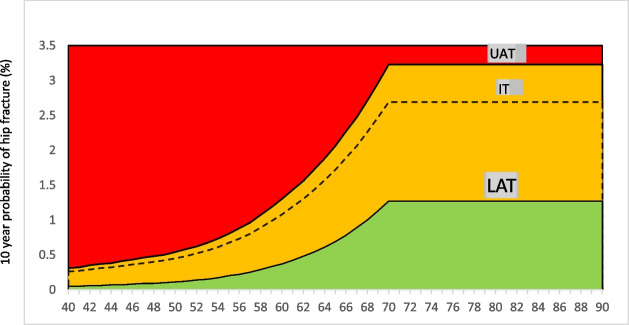
Age-dependent 10-year probability of hip fracture, with the cutoffs for LAT, UAT, and IT. IT, intervention threshold; LAT, lower assessment threshold; UAT, upper assessment threshold

It is recommended to use age-dependent hybrid threshold for risk stratification and management only in postmenopausal women and in men 50 years of age or older (Table 2, Figs. 1 and 2).

Patients can be risk categorized into the following groups.

I. Low risk: if FRAX lies in the green zone that is below the lower assessment threshold (LAT).
II. Very high risk: If FRAX crosses the upper assessment threshold (UAT) (red zone).
III. If FRAX lies between LAT and UAT (orange zone), this is the subset of patients who will need BMD for further risk stratification.
IV. After BMD value, patients will be categorized as low-risk if FRAX lies below the intervention threshold, high risk if it is between intervention and UAT and very high risk if it crosses UAT.
V. Patients with recent onset (12–24 months) hip and spine fractures or multiple fragility fractures are considered a very high-risk group for future fractures.

*Q2. When should I order DXA?*

It is recommended to perform a DXA scan of the lumbar spine and proximal femur for your patients whose 10-year probability of fracture (FRAX) falls within the orange zone—between the lower and upper assessment thresholds. Routine DXA scan without any clinical risk factors or if the FRAX probability is in the green zone below the lower assessment threshold is not recommended. The decision to request a DXA scan in certain special situations (e.g., chronic liver disease, post- and pre-transplant solid or hematological malignancy, breast cancer, patients on aromatase inhibitors, patients on androgen ablation therapy, etc.) depends on the treating physician and can be considered in these special cases.

Combining BMD values with clinical risk factors (FRAX) improve the prediction of hip [40] and fragility fractures and can enhance the sensitivity and specificity of fracture risk prediction.

Patients with a FRAX score in the orange zone are recommended to undergo FRAX calculation after adding the BMD value for risk stratification before starting any treatment.

We recommend doing DXA as a baseline in patients in the very high risk category or any patients who are considered for treatment for future follow-up.

### Investigations

*Q3. What investigations do I need to order before starting anti-osteoporosis medications?*

Basic investigations are recommended for all patients before starting osteoporosis medications; these include complete blood count, a liver function test including albumin, renal function with measurement of GFR, serum calcium and phosphorous, alkaline phosphatase, glucose, vitamin D, parathyroid hormone, and a thyroid function test.

An appropriate medical evaluation (detail history and examination) is indicated in all postmenopausal women and men above 50 years with osteoporosis or at high risk for fracture. Identification of coexisting medical conditions that cause or contribute to bone loss should be done on the initial patient visit. If medical history, physical findings, or laboratory test results suggest causes of secondary osteoporosis, additional laboratory evaluation is warranted, which may include (but is not limited to):

- Serology for suspected celiac disease
- Serum protein electrophoresis and free kappa and lambda light chains for suspected myeloma
- Overnight low dose dexamethasone suppression test for hyperadrenalism
- Serum tryptase and other tests for mastocytosis
- Genetic testing for unusual features that suggest rare metabolic bone diseases
- Serum testosterone level in male patients

### Treatment

*Q4. When should I consider starting pharmacological treatment?*

It is recommended to consider treatment for osteoporosis in the following cases.

- Patients with major fragility fractures (spine, hip, proximal humerus, or distal radius) or multiple fragility fractures, irrespective of BMD
- Ten-year probability of MOF or hip fractures exceeds the age specific upper assessment threshold of Qatar FRAX, irrespective of BMD
- Ten-year probability of MOF or hip fractures exceeds the age specific intervention threshold of Qatar FRAX after adding BMD value

Pharmacological treatment should be offered to all those patients with high and very high risk for fractures. Treatment is broadly divided into two main subgroups based on the mechanism of action. Antiresorptive treatment is targeted at osteoclasts, preventing bone resorption, while anabolic treatment is based on building new bone with a variable effect on bone resorption.

It is recommended to plan for best initial long-term or sequential therapy for each patient based on initial evaluation.

Osteoporosis is a chronic disorder and will need long-term management. Due to the chronicity of the disease and the rare side effects of medications, most of these patients will need sequential therapy rather than using a single agent. The initial assessment and treatment are particularly important in deciding which sequential therapy will benefit the patient the most.

#### Choice of treatment

*Q5. What treatment should I consider in patients with very high risk for fragility fracture?*

Anabolic medication is recommended to be used as the first line in patients with recent onset (12 to 24 months) spine, hip, or multiple fragility fractures. Anabolic medication can be considered an initial therapeutic option if the patient falls into the very high-risk category using FRAX (i.e., crossing the upper assessment threshold).

Studies have revealed that recurrent fractures are more common in the first 2 years of an incident fracture [41]. A study in postmenopausal women demonstrated that 10% of patients sustained recurrent clinical fractures in the first year and 18% had fractures in the next 2 years after the first fracture [42]. These patients need rapid bone building to prevent incident or secondary fractures and will benefit from initial anabolic medication [43].

Teriparatide and romosozumab are the currently available anabolic medications in Qatar. Teriparatide, a recombinant human parathyroid hormone (PTH 1-34) is given as a daily subcutaneous injection. It is an anabolic drug with bone formation more pronounced in trabecular bone, which is reflected in a greater degree of gain in bone mineral density at the spine than at the hip. Teriparatide is effective in reducing vertebral and non-vertebral fractures [44–46]. A randomized control trial in relation to prevention of hip is lacking, but systematic review demonstrated positive effect in reducing hip fracture [47]. A head-to-head trial with risedronate revealed that it is superior to oral bisphosphonate in reducing vertebral fracture [48].

Romosozumab is a humanized monoclonal antibody against sclerostin that has dual mechanism of action, making it unique from other medications. Sclerostin is an endogenous cytokine that inhibits bone formation and promotes bone resorption. The effectiveness of romosozumab in postmenopausal osteoporosis has been demonstrated in multiple trials (ARCH, FRAME, and STRUCUTRE trials) [49–52]. The FRAME trial revealed significantly fewer new vertebral and clinical vertebral fractures in a group of postmenopausal women who received a monthly injection of romosozumab than in those who received placebo, and a similar effect was seen at 24 months when all patients received denosumab at the end of 24 months. The ARCH trial revealed that new vertebral, nonvertebral, clinical, and hip fractures were significantly fewer in women treated with romosozumab followed by alendronate than in those treated with alendronate alone.

*Q6. What treatment should I consider after completing course of anabolic medication?*

It is strongly recommended for patients to commence an antiresorptive medication following the completion of the approved duration of anabolic medication (18–24 months for teriparatide, 12 months for romosozumab).

It is pertinent to mention here that after completing the recommended duration of anabolic treatment, patients should commence antiresorptive treatment, as the effect will be lost if not followed by antiresorptive medications [53–55].

*Q7. What other treatment option can I offer when anabolic agents are not affordable or accessible?*

Denosumab or zoledronic acid is recommended as a 2nd line option for patients with recent spine, hip, or multiple fragility fractures and patients with a very high risk for fracture based on FRAX.

*Q8. What treatment should I consider in patients with high risk for fragility fracture?*

Antiresorptive medications such as oral bisphosphonates, IV zoledronic acid, or denosumab are recommended as initial treatments for high-risk patients. Cost, compliance, patient tolerability, and contraindications to medication should be considered before starting antiresorptive medication.

Bisphosphonates are the oldest medications used in the management of osteoporosis. It is available in oral and IV formulations. Alendronate, risedronate, and ibandronate are the commonly used oral medications, and ibandronate and zoledronic are available in IV formulation. Bisphosphonates are effective in the treatment of postmenopausal osteoporosis, osteoporosis in men, and steroid-induced osteoporosis. All bisphosphonates [56–67] are effective in reducing vertebral, hip, and nonvertebral fracture risk apart from ibandronate, which is only effective in vertebral fracture risk reduction. It is recommended to reassess these patients within 3 months for tolerability and compliance, especially patients on alendronate.

Denosumab, a fully human monoclonal antibody, is one of the most potent antiresorptive medications. It binds to the receptor activator of the nuclear factor kappa-Β (RANK) ligand (RANKL) preventing its interaction with the RANK receptor on osteoclast, inhibiting bone resorption. Denosumab [68–70] is effective in reducing the incidence of vertebral, non-vertebral, and hip fractures. An increase in bone mineral density has been observed in all major sites after treatment with denosumab. In contrast to bisphosphonates, where gain in hip bone mineral density plateaus after 3 to 5 years of treatment, continuous BMD gain was observed with denosumab treatment beyond 5 years of treatment. Data up to 10 years of denosumab use is available without any significant safety concerns [71]. Denosumab can be used as initial antiresorptive therapy in high-risk patients and in patients who fail to respond to other antiresorptive medications.

It is recommended to make a long-term plan with patient if denosumab is considered for treatment. Denosumab should be administered every 6 months and should not be stopped without switching to bisphosphonate.

Denosumab therapy should not be stopped abruptly, as it can lead to rapid loss of bone mineral density and increase the incidence of multiple vertebral fracture [72–75]. Patient should be commenced on bisphosphonate 6 months post-last dose of denosumab if planned to discontinue it.

*Q9. When can I consider selective estrogen receptor modulator (SERM) in patients with high risk for fragility fracture?*

Raloxifene can be considered as an alternative antiresorptive medication in high-risk young postmenopausal women if the aim is reducing vertebral fracture risk. It can also be considered for treatment of vertebral fracture when all other options are not feasible.

Raloxifene belongs to the group of SERMs with specific estrogen binding affinity. It is approved for the treatment of postmenopausal osteoporosis. Studies have revealed that it is effective in reducing the incidence of vertebral fracture in patients with and without a previous history of fragility vertebral fracture [76, 77]. Efficacy in terms of reducing non-vertebral and hip fractures is not demonstrated in clinic trials. It can be considered in patients with high risk for fracture as an alternative to bisphosphonate and denosumab. Raloxifene should not be used in childbearing women, in cases of unexplained uterine bleeding, severe hepatic and renal impairment, or in women with a history of venous thromboembolism.

### Monitoring and duration

*Q10. How should I monitor a patient receiving treatment?*

DXA is recommended every 1–2 years to assess the response to therapy. Bone resorption and bone formation markers can be considered for monitoring the patient’s response and adherence to medication. Bone markers should not be used for the diagnosis of osteoporosis.

In treated patients, if BMD decreases significantly, patients should be evaluated for noncompliance, secondary causes of osteoporosis, or use of medications that might cause bone loss. Ideally, BMD monitoring should occur at the same facility, using the same DXA machine and, if possible, the same technologist as the previous DXA, and should involve the same ROIs for both the spine and hip.

Monitoring bone turnover markers provides valuable information about the rate of bone formation and resorption, helping physicians evaluate the impact of the treatment on bone health and patients’ adherence to treatment [78–82].

Due to the quick response to therapy, physicians can use bone markers as early as 3 months rather than waiting 1–2 years for a DXA scan. Bone markers should be measured initially and then 3–6 months after the start of osteoporosis treatment.

It is important to collect serum samples in the morning (7.30–10.00 h) and after an overnight fast for measurement of bone markers. Multiple factors can affect the serum blood sample of bone markers as shown in Table 3.

**Table 3:** Factors affecting bone marker levels

	Effect	Recommendation	Importance
Circadian rhythm	High BTM concentrations at night and early morning, lowest in the afternoon	Collect serum samples in the morning (7.30–10.00 h)	High
Food intake	Decrease in BTMs, especially bone resorption markers (about 20–40%) after food intake	Collect samples of bone resorption markers after overnight fast	High
Medications oral GCs	Rapid and dose-dependent decrease in bone formation markers	Consider dose of oral GCs	High
Fracture	BTMs increase after fracture, with maximum effect 2–12 weeks, but remains elevated up to 52 weeks	Limits evaluation in patients with recent fracture	High
Menopause	BTMs increase at the time of the final menstrual period	Use reference intervals considering menopausal status	Moderate

*BTM* bone turnover marker, *GC* glucocorticoid

1. Bone markers to measure:

- Bone resorption marker: CTX
- Bone formation marker: PINP, bone alkaline phosphatase

2. Timing of measure:

- Initially (before starting treatment)
- 3–6 months after the start of therapy

3. Purpose of measurement:

- Assess the effectiveness of the treatment.
- Evaluate adherence to therapy

### Drug holiday

*Q11. When should I consider drug holiday in patients on BP’s?*

It is recommended to consider drug holiday for patients on bisphosphonates (5 years after oral and 3 years after IV).

Studies have revealed that compared to the occurrence of fragility fractures after discontinuation of medication, skeletal adverse effects like atypical femoral fractures are still rare in patients using long-term BPs [75, 83]. It is very important to carefully select patients for drug holidays. Patients without fragility fracture, whose T-score at the total hip is ≥ − 2.5 or who are not on steroid therapy, can be considered for drug holidays after 5 years of oral and 3 years of IV BP’s. The patient should be reevaluated in 1 to 2 years with DXA and should be offered medication if BMD declines significantly. Contrary to this, for patients who remain at high risk of fracture (patient with a history of fragility fracture, T-score at total hip is still less then − 2.5) after 3 years of (intravenous) or 5 years of oral BP treatment, it is recommended to switch to denosumab or anabolic treatment based on risk stratification.

*Q12. What additional measures should I provide to maintain healthy bones?*

Several lifestyle modifications play a crucial role in improving musculoskeletal integrity and balance and preventing future fractures. This advice should be given to all who are being assessed for bone health and fracture risk.

All postmenopausal women and men age ≥ 50 years should obtain an adequate intake of dietary calcium. Controlled clinical trials have demonstrated that the combination of supplemental calcium and vitamin D can reduce the risk of fracture [84–87]. Vitamin D deficiency is common in patients with osteoporosis and hip fractures [88]. The evidence regarding the direct impact of vitamin D supplementation on fracture prevention has been mixed. There is little evidence that vitamin D supplementation alone reduces fracture incidence, although it may have a role in reducing falls [86, 87]. A daily calcium intake of between 800 and 1200 mg, ideally taken through dairy products, is recommended. Calcium supplementation is required in cases of inadequate dietary intake. A daily dose of 800 IU of cholecalciferol should be advised for postmenopausal women at increased risk of fracture or exhibiting evidence of vitamin D insufficiency. Regular weight-bearing exercise tailored to the needs and abilities of an individual patient is recommended. Smoking cessation should be discussed with the patient, as its use is detrimental to the skeleton as well as to overall health. Obtaining a history of falls is an essential component of assessing individuals at increased risk of fractures. Falls can lead to fractures, and individuals who have a history of falls are at a higher risk of fracture in the future. Individuals identified as having an increased risk of falls should undergo further assessment to identify specific factors contributing to their fall risk
